# A game of substrates: replication fork remodeling and its roles in genome stability and chemo-resistance

**DOI:** 10.15698/cst2017.12.114

**Published:** 2017-12-05

**Authors:** Julia Sidorova

**Affiliations:** 1 Department of Pathology, University of Washington, Seattle, Washington, USA.

**Keywords:** DNA, replication fork, S phase, end resection, human, vertebrate, chemotherapy

## Abstract

During the hours that human cells spend in the DNA synthesis (S) phase of the cell cycle, they may encounter adversities such as DNA damage or shortage of nucleotides. Under these stresses, replication forks in DNA may experience slowing, stalling, and breakage. Fork remodeling mechanisms, which stabilize slow or stalled replication forks and ensure their ability to continue or resume replication, protect cells from genomic instability and carcinogenesis. Fork remodeling includes DNA strand exchanges that result in annealing of newly synthesized strands (fork reversal), controlled DNA resection, and cleavage of DNA strands. Defects in major tumor suppressor genes BRCA1 and BRCA2, and a subset of the Fanconi Anemia genes have been shown to result in deregulation in fork remodeling, and most prominently, loss of kilobases of nascent DNA from stalled replication forks. This phenomenon has recently gained spotlight as a potential marker and mediator of chemo-sensitivity in cancer cells and, conversely, its suppression - as a hallmark of acquired chemo-resistance. Moreover, nascent strand degradation at forks is now known to also trigger innate immune response to self-DNA. An increasingly sophisticated molecular description of these events now points at a combination of unbalanced fork reversal and end-resection as a root cause, yet also reveals the multi-layered complexity and heterogeneity of the underlying processes in normal and cancer cells.

## INTRODUCTION

The three billion base pairs of the human genome are replicated in about six to eight hours of the S phase of the cell cycle. At the height of S phase, there are hundreds of replication forks running through chromosomes, each carrying on its three-way DNA junction anywhere from dozens to perhaps low hundreds of proteins that service every aspect of chromatin duplication. When replication forks experience disruptions to their normal mode of operation, which manifests as forks’ stalling or slowing, the condition is referred to as *replication (*or* replicative) stress*. Among classic causes of replication stress are DNA damage during S phase and unbalanced or reduced levels of intracellular nucleotide pools, both of which can be elicited by exogenous agents such as chemotherapy drugs. Other recognized causes include conflicts with transcription, epigenetic abnormalities, and defective cell cycle regulation. Prolonged stalling of forks during stress can cause fork collapse, i.e. DNA damage and loss of protein machinery of the fork, which can jeopardize complete duplication of the genome. Replication stress is considered one of the major drivers of genomic instability as well as premature senescence and carcinogenesis. For in-depth review of these topics, see 1,2,3,4,5,6,7,8,9.

Eukaryotic cells have evolved elaborate mechanisms to buffer replication fork activity against stress. These mechanisms can be divided into two categories: those that enable bypass of damage, and those that allow stable pausing and on-demand resumption of replication once the obstacle is cleared or conditions are normalized. It is the latter group that we will focus on in this review. For additional discussion of the subject, we recommend these excellent recent reviews 10,11,12,13,14.

### Stalled forks are remodeled

It is helpful to distinguish three processes that can occur at a stalled replication fork and involve DNA strand separation/annealing, degradation, and breakage. These are, respectively, fork reversal, resection, and collapse (for the mechanisms engaged at moving forks, i.e. translesion synthesis (TLS) and repriming, we recommend a recent review 15). We will use the term fork *remodeling* to refer to these processes. Evidence suggests that reversal and resection are actively regulated, and not only are compatible with but in fact enable stabilization of forks and contribute to their ability to resist disruptions of DNA synthesis (see next section). Fork collapse may be viewed as an exhaustion or breakdown of fork remodeling over time. Likely however, it is an actively triggered pathway of last resort initiated by cleavage of one arm of a fork by the MUS81 endonuclease 16,17,18,19. Collapsed forks may be resolved into unreplicated parental strands or repaired and reactivated 20. Recent findings implicated Break-Induced Replication (BIR, 21) in reactivation of collapsed replication forks in humans 22, and demonstrated MUS81-dependent BIR in S phase 19 as well as in mitosis 23,24. In the interest of space, this review will predominantly focus on the mechanisms that preserve an active fork collapse-free. For an excellent overview on fork remodeling, see 25.

**Figure 1:**
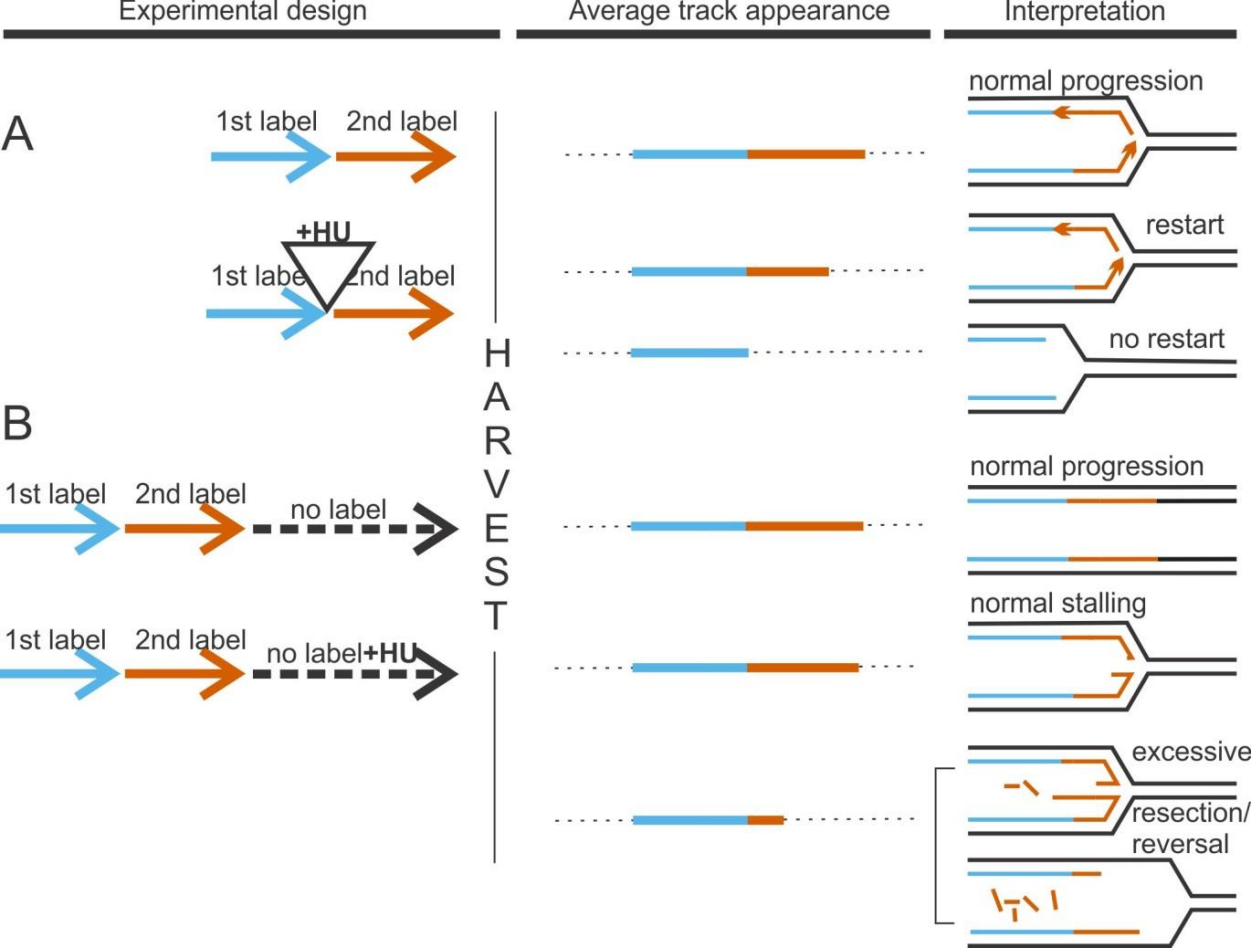
FIGURE 1: DNA fiber analysis detects faulty fork activity. In a "classic" experiment to measure fork restart **(A)**, cells are sequentially pulse-labeled for 15-30 min each with two nucleoside analogs, typically, chloro-deoxyuridine and iodo-deoxyuridine. In test samples, incubation with a fork-arresting dose of hydroxyurea (HU), a ribonucleotide reductase inhibitor, is introduced between the labels. Failure to restart after HU manifests as preponderance of tracks containing only 1^st^ label. One way to measure degradation of nascent strands at stalled forks **(B)**, is to follow sequential pulse-labeling with two labels by a label-free chase (usually 4-6hrs). In test samples, HU is introduced during this time. Extensive loss of nascent DNA manifests as shortening of 2^nd^ label segments in tracks labeled prior to HU addition.

### Reversal/restoration cycle helps to maintain forks’ ability to resume replication

**Figure 2:**
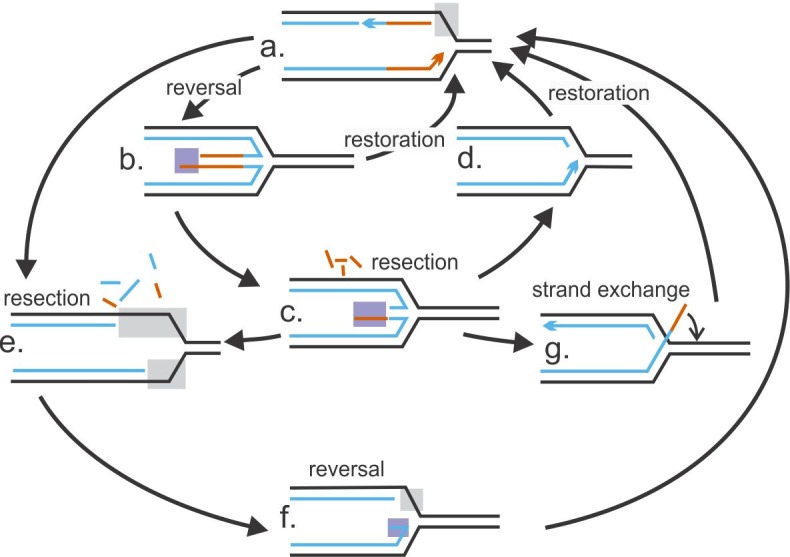
FIGURE 2: Fork remodeling as interlocked cycles of reversal and resection. A framework to visualize remodeling of stalled forks as connected, dynamic states that are characterized by prevalence of reversal or restoration, and resection or protection. Gray rectangles mark exposure of parental ssDNA and purple rectangles - nascent ssDNA. Both can be used as markers of fork state.

Reversed forks (a.k.a. regressed forks, or "chicken feet", **Figure 1B, 2**), in which two nascent strands pair with one another to form a forth branch have first been observed by electron microscopy (EM) in the preparations of genomic DNA decades ago 26. More recently, their biological relevance came into focus with the studies from Lopes and Vindigni labs, which positively correlated presence and abundance of reversed forks in human genomic DNA with the ability of forks to resume replication *in vivo* after treatment with several different fork-interfering agents 27. Lopes lab demonstrated that the RAD51 protein, a single-stranded (ss) DNA-binding RecA homolog of eukaryotes first known for its strand exchange activity in homologous recombination, is required for fork reversal 28 (for a recent review of RAD51, see 29). RAD51 is also required for resumption of replication by stalled forks, which supports the idea that reversal preserves forks’ activity 30,31. RAD51 probably does not execute reversal but rather, stabilizes the reversed fork 32. However, several proteins can directly stimulate reversal of forks (**Table 1**). *In vitro*, the RAD54 DNA translocase 32,33, RECQ helicases WRN and BLM 34, and the FANCM helicase 35 showed this activity. The proteins FBH1, SMARCAL1, ZRANB3, and HLTF, as well as the MMS22L/TONSL complex were shown to reverse forks both *in vitro* and *in vivo* 19,36,37,38,39,40,41,42,43,44.

**Table 1:** TABLE 1. Proteins capable of fork reversal and/or restoration. See main text for relevant references.

**Protein**	**Enzymatic activity**	**Fork remodeling contribution**	**Activity on RAD51**
BLM	DNA helicase	Reversal/restoration	Filament destabilization
FANCM	DNA helicase	Reversal, lagging strand junction-gapped	
FBH1	DNA helicase	Reversal	Filament dissolution
HLTF	DNA helicase	Reversal, leading strand junction-gapped or not, restoration not shown	
	E3 Ubiquitin ligase		
MMS22L/TONSL	None noted	Reversal	Filament formation
	Adaptor/scaffold		Reduces binding to dsDNA
RAD54	DNA translocase (not a helicase)	Reversal/restoration (branch migration)	Displacement of RAD51 from dsDNA (end stage HR)
			Stabilization of RAD51/ssDNA
RECQ1	DNA helicase	Restoration	
SMARCAL1	Annealing helicase	Reversal of leading strand junction-gapped forks, also restoration of reversed forks	
WRN	DNA helicase	Branch migration	Deficiency associated with reduced loading of RAD51
	exonuclease	reversal/restoration	
ZRANB3	Annealing helicase and endonuclease	Reversal and restoration	D-loop dissolution
		ssDNA-gapped fork is preferred	

**Figure 3:**
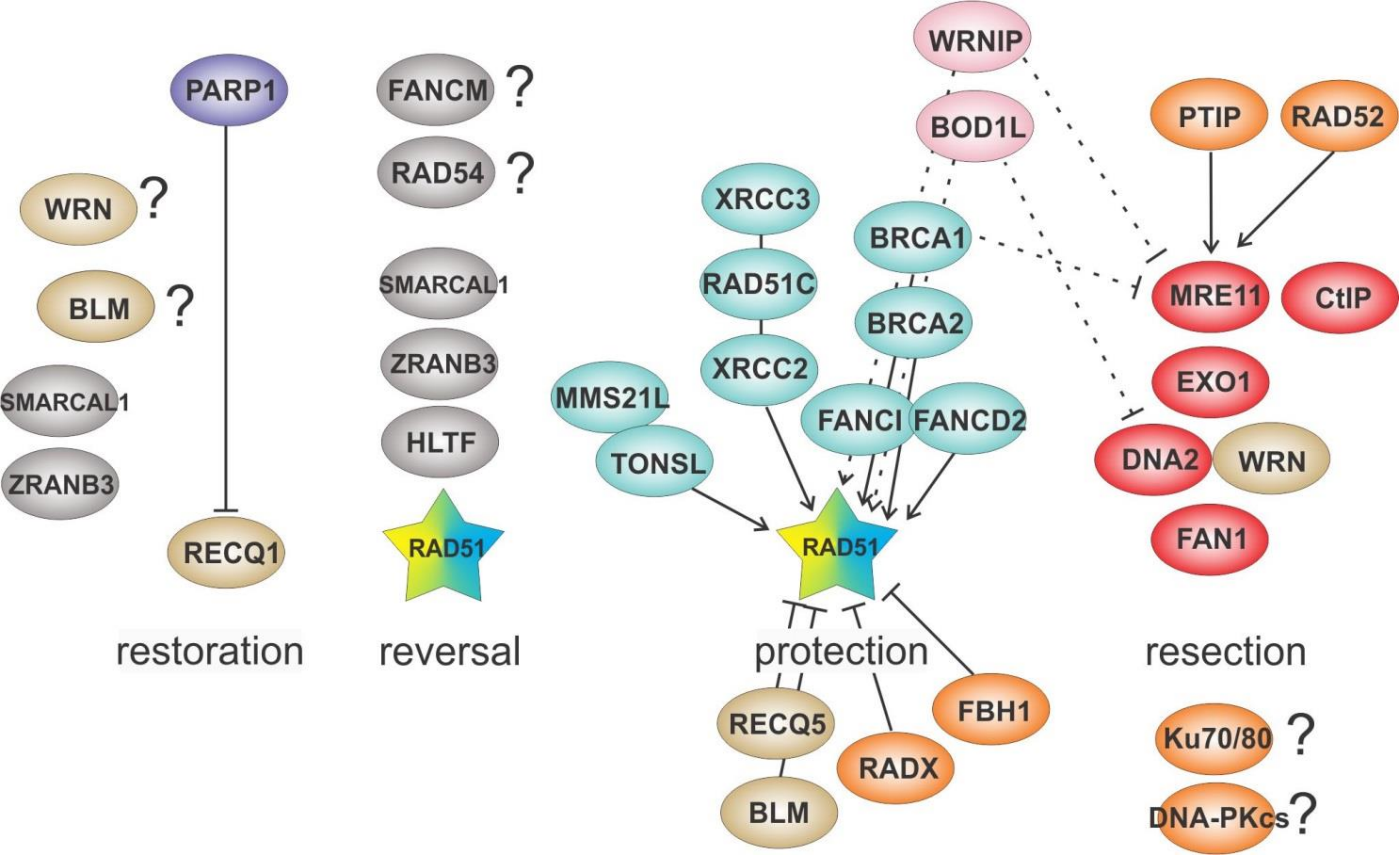
FIGURE 3: An overview of proteins that mediate fork reversal/restoration and resection/protection. Functional relationships between proteins are indicated by solid lines, and dashed lines indicate likely inferences.

FBH1 is a 3’-5’ DNA helicase capable of targeting its binding partners for ubiquitination 45. SMARCAL1 and ZRANB3, two related members of the SNF2 family of ATP-dependent DNA helicase-like proteins, as well as their more distant relative HLTF, are required for stalled fork restart (see **Figure 1A** and **BOX1** for technical background) 36,46,47,48. SMARCAL1 is mutated in in a subset of Schimke’s Immunoosseous Dysplasia (SIOD) cases, and this mutation also abolishes SMARCAL1 fork reversal activity *in vitro* and triggers fork collapse and DNA damage *in vivo*, further supporting the notion that fork reversal is a protective mechanism 47,49. SMARCAL1, ZRANB3, and HLTF show differential fork substrate preferences with regard to the presence and location of ssDNA gaps, and are hypothesized to have non-redundant roles in the cell. Also, only SMARCAL1 and ZRANB3 but not HTLF are able to perform the reaction opposite to fork reversal, i.e. fork restoration. For further detail on the roles of these proteins at forks, see **Table 1** and 50,51; also, **Figure 3**.

**Box1:**
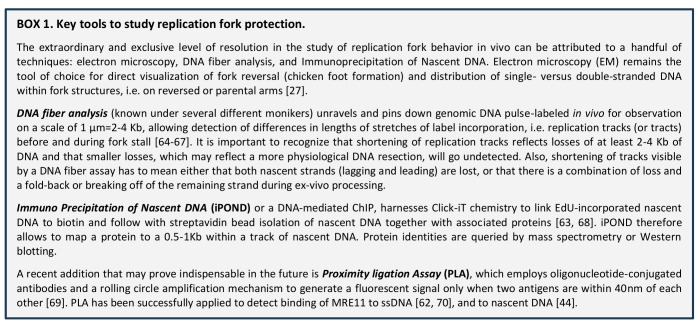


While failure to reverse forks has deleterious consequences for fork activity and genome stability overall, studies find that excessive fork reversal, e.g. due to overexpression of SMARCAL1 or RAD51, can also result in DNA damage and threaten genome stability 52,53,54,55. This suggests a notion that fork reversal is a "double-edged sword", and needs to be balanced. Indeed, an additional control over fork reversal has been identified in poly(ADP-ribose)-polymerase 1 (PARP1) and the RECQ1 helicase. PARP1-dependent PARylation of DNA and proteins is involved in coordinating responses to DNA damage and replication disruptions 56,57. Activated PARP1 was shown to stimulate accumulation of reversed forks *in vivo* 58. The mechanism behind this phenotype was elucidated by Vindigni and colleagues, who showed that PARP1 does not directly promote fork reversal. Instead, it inhibits the RECQ helicase family member RECQ1, whose function is to preferentially restore reversed forks to their original three-armed configuration *in vitro* and *in vivo *59. In particular, under conditions of fork arrest/slowing, an increase in reversed forks was suppressed by inhibition of PARP1, but strikingly, not in the absence of RECQ1. Taken together, the data suggest that the duration and extent of fork reversal *in vivo* is orchestrated by counteracting activities and is regulated by a master-switch PARP1 that prevents premature restoration of a reversed fork.

### Nascent strand resection at stalled forks

Schlacher and colleagues in the Jasin lab were the first to put degradation of nascent DNA at stalled forks in the spotlight 60,61. Using DNA fiber assays, they observed loss of kilobases of newly-synthesized DNA from forks stalled by the nucleotide depleting drug hydroxyurea (HU), which manifested as pronounced shortening of replication tracts laid down immediately *prior to *fork arrest (**Figure 1B** and **BOX1**). This phenotype was present in cells defective for breast cancer susceptibility genes BRCA1 and BRCA2, or Fanconi Anemia genes FANCD2, or FANCA, but not in the wild type cells. The authors implicated the MRE11 nuclease known for its role in DNA end resection during double strand break (DSB) repair, in the loss of nascent DNA at forks. Moreover, they provided evidence that nucleolytic attack by MRE11 was upregulated without BRCA2 because no ssDNA/RAD51 filament was there to protect the fork. In particular, BRCA2 C-terminal domain involved in stabilizing RAD51 filament on ssDNA was critical for preventing degradation. Overexpression of BRC4 domain of BRCA2, which binds RAD51, or of the K133R mutant of RAD51, which forms hyper-stable filaments, respectively, enhanced and suppressed nascent strand degradation.

The C-terminus mutant of BRCA2 that failed to protect forks from degradation was able to maintain homology-dependent DNA repair, suggesting, crucially, that this function of BRCA2 is at least somewhat separate and different from its DSB repair function. Schlacher *et al*. thereby referred to this distinct pathway as *fork protection* to highlight the protective functions of RAD51 and BRCA/FANC genes. Notably, BRCA2 absence did not inhibit fork restart after HU removal, though it increased DNA damage and genomic instability associated with HU treatment. Though BRCA2 C-terminal mutants were later found defective in fork restart 42,71, it has been observed time and again that resection does not necessarily result in a fork that is unable to restart replication, e.g. see 19,72.

Overall, stalled fork condition emerged as a counterbalance of protective activity of RAD51 and nucleolytic activity of MRE11, a kind of a battle between a hero and an anti-hero. However, several observations complicated the picture. For example, RAD51 depletion does not always result in upregulated nascent strand degradation at stalled forks (e.g. compare 28,73,74). MRE11, on the other hand, has been shown to have a fork protective effect: without MRE11, fork stalling is associated with increased DNA damage, and forks are less capable of resuming replication 75. MRE11 is recruited to stalled forks by PARP1, and this is a part of the fork-protective role of PARP1 76.

In addition, Hashimoto *et al*. 77 observed both MRE11-dependent and independent ssDNA gap accumulation in replicating DNA upon loss of RAD51 in Xenopus egg extracts. Also, a publication by the Vindigni group demonstrated nascent strand degradation in U2OS cells upon depletion of RECQ1 and with the proficient BRCA1 and BRCA2 genes 73. This degradation was executed by the DNA2 endonuclease in conjunction with the WRN helicase and surprisingly, *did not* involve MRE11. Moreover, depletion of RAD51 did not exacerbate this degradation but, on the contrary, *prevented* it. These observations suggested a more elaborate view of fork protection, as we will discuss below.

### Fork remodeling as interlocked cycles of reversal and resection

To begin, resection and reversal can be viewed as interlocked, mutually constraining fork remodeling processes (**Figure 2**). The extent of resection or reversal as well as prevalence of one over the other may vary depending on relative abundances and activities of the involved proteins. In theory at least, reversal may precede and/or follow resection.

The timing of appearance and prevalence of single-stranded (ss)DNA at forks is a critical readout of fork reversal and resection. *Parental* ssDNA (gray boxes in **Figure 2**) can be initially exposed due to failure of DNA synthesis and excessive unwinding ahead of the fork by the replicative helicase, and then expand due to resection. Appearance of *nascent* ssDNA (purple boxes in **Figure 2**) strongly suggests reversal with subsequent resection. RAD51, MRE11, and other proteins may act either on the single-stranded portion of an extruded nascent strand duplex, i.e. the "forth" arm of a reversed fork (at structures **b** and **c**, **Figure 2**), or on the parental DNA over a single-stranded gap (structure **e**); the latter mode may be followed by reannealing of the parental duplex (structure **f**). Techniques such as Proximity Ligation Assay (see **BOX1** for detail) can now begin to distinguish between these scenarios and will help delineate specific sequences of events in individual model systems. The order or prevalence of reversal versus resection may be the reason the phenotype of RAD51 deficiency can be either protective or sensitizing for resection. That is, RAD51 depletion will suppress resection if RAD51 acts only at reversal stage (i.e. at **a →b** path in **Figure 2**), and resection is strictly dependent on prior reversal (i.e. **a →e **path is not active) 19,62,73. In the next sections we will use this framework as a guide to undertake an in-depth analysis of the rapidly growing body of data on the interplay between fork resection and reversal.

## THE KEY PLAYERS AND PROCESSES

**Figure 3** lists the proteins that contribute to remodeling of stalled forks. Some of these proteins were already mentioned in the previous sections. These and other proteins will be the subject of the discussion below.

### MRE11 and other nucleases

Historically, many proteins that are active on stalled forks were first recognized for their roles in the repair of two-ended DSBs in DNA. MRE11 is no exception. Much of what is known about MRE11 at the molecular level comes from the studies of DSB end processing in the context of competition between homologous recombination (HR)-mediated repair and non-homologous end joining (NHEJ). MRE11’s ssDNA endonuclease activity initiates resection by introducing a nick internal to a DSB end. The nicked strand is then degraded in the 5’-3’ direction by the EXO1 exonuclease and in the 3’-5’ direction by MRE11’s exonuclease activity 78,79, which is the activity inhibited by the commonly used MRE11 inhibitor mirin. For long-range resection, MRE11 and EXO1 are joined by the DNA2 endonuclease assisted by the RECQ helicases BLM or WRN 80. Neither EXO1 nor DNA2 complexes are strictly dependent on MRE11 for their recruitment to DSBs. Resection exposes a ssDNA 3’ overhang, onto which RAD51 is loaded for subsequent homology search and strand exchange. For further detail, see 81,82.

The extent of contribution of MRE11 exonuclease to resection appears to vary between experimental systems 83,84. A recent report suggests that MRE11 exonuclease is a minor contributor to resection but, along with CtIP, is key to displacing Ku70/80 from DNA ends 85. Without such displacement, RAD51 loading onto ssDNA is reduced 85, and processing of DNA ends by EXO1 is blocked 83. In sum, MRE11 commits a DSB to resection, counteracts resection inhibitors such as Ku70/80, but also facilitates loading of RAD51 on ssDNA.

At replication forks, the forth arm that forms upon fork reversal bears resemblance to a one-ended DSB, and is expected to be a substrate for resection in a manner analogous to the above. Some evidence is also consistent with MRE11 targeting nascent DNA ends that flank ssDNA gaps within newly replicated parental/daughter DNA duplexes 42. In contrast to a DSB however, a stalled fork may not require MRE11’s non-redundant function as an initiator of resection, because it already contains ssDNA nicks originating from the Okazaki fragments. In addition, unlike at DSBs, other resection-capable proteins, i.e. DNA2, BLM, and WRN are already present at forks, assisting in normal replication 16,31,86,87,88,89. These functions may help explain why MRE11 may have a negligible input into resection at stalled forks at least in some cases 62,73. On the other hand, unlike at DSBs, MRE11, which is not capable of performing long-range resection, may be able to degrade more DNA at forks during repeated cycles of resection - reversal - resection.

### BRCA1 and BRCA2: many roles versus one?

At DSBs, BRCA1 both promotes and limits strand resection (for review, 90,91,92. On the one hand, it assists in loading MRE11 and stimulating its endonuclease activity via CtIP 93. BRCA1 also facilitates recruitment of EXO1 and suppresses DNA-PK activation 94. One the other hand, one of BRCA1 complexes, BRCA1-A, also appears to inhibit resection once it has exceeded a certain distance from the original break site. Since MRE11’s role in initiating resection at forks may be dispensable, it is the restraining function of BRCA1 that may be prominent, explaining the increased resection and a higher level of MRE11 on nascent DNA in BRCA1-deficient cells. Lastly, via PALB2, BRCA1 also aids BRCA2 in loading RAD51 onto single stranded DNA 95. The importance of BRCA1 at forks is underscored by a recent finding that BRCA1 is haploinsufficient for suppressing nascent strand degradation at forks 96.

Compared to many facets of BRCA1, the role of BRCA2 at forks seems straightforward enough: along with PALB2 and RAD51 paralogs it directs formation of the RAD51/ssDNA filament. BRCA2 also discourages binding of RAD51 to dsDNA 97,98, which may buffer RAD51 activity, since excess RAD51 and its resulting association with dsDNA may be inhibitory to strand exchange. Even so, some loading of RAD51 may occur independently of BRCA2, for example by MMS22L/TONSL 40. Recent findings in the Xenopus egg extract system indicate that BRCA2 loss is associated with ssDNA gaps at and behind the forks even in the absence of exogenous fork-arresting agents 42; and slowed fork progression was noted in a BRCA2-deficient human cell line 99. It is possible that these defects in fact predispose forks in BRCA2-deficient cells to reversal upon stalling.

It should be noted that recent work also points at mechanistically important differences between BRCA1 and BRCA2 in the ways these proteins contribute to the fate of forks *beyond *the protection/resection cycle. BRCA2 deficiency has been associated with *increased* MUS81-mediated fork cleavage followed by BIR in S phase (during recovery from HU-mediated stalling) 19, or in mitosis (during an unperturbed S phase) 99. On the other hand, BRCA1 deficiency was shown to *suppress* mitotic MUS81-coupled BIR after fork stalling 100. These differences will have to be incorporated into the field’s developing view of BRCA-deficient cell survival and mutagenesis upon replication stress.

### RAD51 versus the nucleases: reversal, protection, resection?

RAD51 is recruited to a stalled fork and is one of the factors that reverses it or stabilizes an already reversed fork 28. Appearance of RAD51 at a stalled fork occurs at about the same time if not slightly before MRE11 63. MRE11 recruitment to a fork is promoted by PARP1 that also promotes fork reversal 59,76. Thus, it is a reversed fork that can be a substrate for nucleolytic degradation by MRE11. In this scheme RAD51 is therefore upstream of MRE11 and provides a substrate for it. At the same time, RAD51 can limit the activity of MRE11 and other nucleases on this substrate, and if RAD51 is not there, the reversed fork is over-digested to the extent that a regressed arm disappears. Therefore, RAD51 can also act downstream of MRE11, and it is in this sense that MRE11 provides a substrate for RAD51, which agrees with the finding that inhibition of MRE11 exonuclease activity can reduce the amount of RAD51 loaded at HU-stalled forks 63. Several new papers now provide strong evidence in support of this model. Using a Xenopus egg extract system 42 or several human cell lines 19,43,44 four labs have shown that upon depletion of BRCA2 or BRCA1, EXO1 and MRE11 non-redundantly degraded nascent DNA at forks, and strikingly, this degradation could be alleviated by reducing fork reversal. This was achieved when RAD51, or the SNF2 family members SMARCAL1, ZRANB3, or HLTF, were depleted or mutated. Thus, at least in the context of BRCA1/2 inactivation, reversed forks are a predominant substrate for MRE11 and EXO1 exonucleolytic degradation of nascent DNA and RAD51 is critical for fork reversal.

The dual role of RAD51 before/during and after fork reversal was supported by two observations. Mijic *et al*. found that the T131P mutation of RAD51 that prevents it from forming a stable nucleofilament 101 is a separation of function mutation: it retains RAD51’s ability to promote/stabilize fork reversal but fails to protect reversed forks from MRE11 43. Taglialatela *et al.* observed that B02 inhibitor of RAD51, which blocks its binding to DNA 102, elicited increased nascent strand degradation that was suppressed by depleting SMARCAL1 44. This suggested that B02 blocked fork protecting function of RAD51 downstream of fork reversal.

In sum, there is now a very strong case for a reversed fork as an entryway for nascent strand degradation by MRE11 and EXO1 (**Figure 3**). That said, the possibility that nascent strands can also be degraded in non-reversed forks by MRE11 or other nucleases is not ruled out, particularly for the case of BRCA1/2-proficient cells. If so, such nucleolytic activity will expose parental ssDNA that can serve as a substrate for RAD51, as it does during DSB repair. The final resolution of the question will require data on when parental and/or nascent ssDNA accumulate during fork remodeling, and which of these ssDNA are bound by RAD51, MRE11, and other proteins.

### The nuclease crosstalk and the curious case of WRN

As we have seen, MRE11 and EXO1 are demonstrably active in fork resection in the BRCA1/2-deficient backgrounds. In contrast, Lemacon *et *a.l showed that DNA2 is not involved in BRCA1 or BRCA2-deficient cells 19. Similarly, we found that in a BRCA1-deficient background of an ovarian cancer cell line, depleting WRN does not suppress upregulated resection, indicating that WRN is not involved in it (Sidorova, unpublished). Chaudhuri *et al*. made a similar observation in BRCA2-deficient cells, though also found that DNA2 was in fact involved (perhaps assisted by another RECQ helicase), and MRE11 and DNA2 were epistatic with respect to nascent strand degradation, suggesting a crosstalk between these nucleases 103.

On the other hand, DNA2/WRN and not MRE11 or EXO1 carried out resection in cases where nascent strand degradation was upregulated in *BRCA-proficient* cells 73. Together, these findings suggest a possibility that BRCA1/2 status may influence involvement of the nucleases that degrade nascent DNA.

A study by Iannascoli *et al*. adds a helpful hint to this puzzle 62. In particular, the WRN exonuclease-dead mutant E84A but not helicase or null mutants appears to trigger nascent strand degradation in BRCA-proficient cells and accumulates excess *nascent ssDNA *in SV40-transformed human fibroblasts (consistent with a reversed fork structure **c** in **Figure 2**). To our knowledge, this is the demonstration of restrained resection limited to the reversed arm. Moreover, both nascent ssDNA and nascent strand degradation phenotypes were suppressed by depletion of MRE11 (or EXO1) or by inhibition of MRE11 exonuclease activity. One possibility suggested by these data is that there is a regulatory link connecting DNA2/WRN to MRE11, EXO1 at stalled forks. Here it is worth mentioning that WRN has been associated with *promoting* RAD51 binding to stalled forks in two studies 72,104.

**Figure 4:**
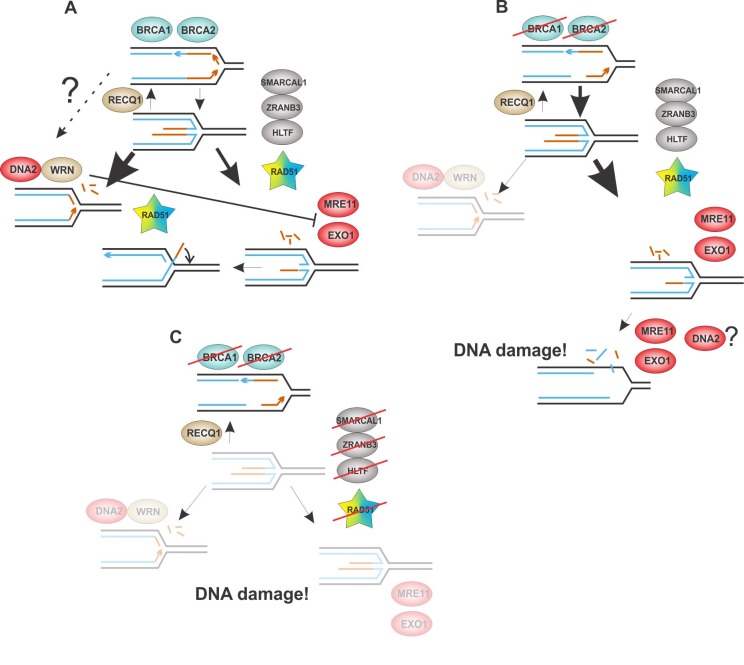
FIGURE 4: A model for two interlinked branches of fork resection and their alterations upon mutational loss of the involved proteins. In a wild type cell the MRE11, EXO1 branch may be less prevalent than the DNA2/WRN branch, depending on the cell background. An inhibitory feedback from WRN to MRE11 may contribute to this arrangement. Fork resection may be limited by restorative branch migration of the fork junction, or reannealing of the 3’ overhang in a D-loop-like strand exchange **(A)**. In BRCA1 or BRCA2-defective cells, the MRE11, EXO1 branch dominates and WRN is no longer a contributor. Fork resection proceeds beyond the four-way junction, either directly, or via cycles of reversal/resection, leading to DNA breakage either directly or via segregation of under-replicated DNA **(B)**. Inhibition of fork reversal suppresses fork resection but also decreases stalled fork stability and ability to resume replication. DNA damage may result from fork collapse and segregation of under-replicated DNA and is exacerbated by BRCA1 or BRCA2 deficiency **(C)**. Faded areas in the cartoon indicate pathway inactivity. See text of the sections "RAD51 versus the nucleases: reversal, protection, resection?" and "The nuclease crosstalk and the curious case of WRN" for a detailed discussion.

Based on the data reviewed in this and previous sections, we therefore hypothesize that there are two, potentially interconnected, pathways that are capable of resecting nascent DNA at reversed forks (**Figure 4**). Their relative prevalence may depend on the BRCA status and/or yet unknown genetic determinants that define the balance between fork reversal/restoration. Once DNA2/WRN are engaged, this inhibits MRE11 and EXO1. This model thus depicts a scenario very different from the HR-mediated DSB repair and will require the support of additional data for its validation.

## FACILITATOR AND RESTRAINER PROTEINS ADD A LEVEL OF COMPLEXITY TO FORK REMODELING 

In this section we will review the protein factors that facilitate or counteract RAD51 and MRE11 at stalled forks, and consider what these proteins can tell us about the dynamic interplay of reversal/restoration and protection/resection processes during fork remodeling, and their potential plasticity in different cells.

### PTIP and RAD52 as facilitators of MRE11

In DSB repair, PTIP works as a facilitator of NHEJ counteracted by BRCA1 105. A recent report suggests that at DSBs PTIP recruits Artemis (DCLRE1C), an endonuclease that cleaves DNA at ss/dsDNA junctions and can trim 5’-3’ overhangs and well as attack ss gaps, generating DNA ends compatible with NHEJ 106. Consistent with these findings, loss of PTIP in BRCA1-deficient cells renders them PARP inhibitor- and cisplatin-resistant and partially rescues end resection at DSBs 105. Remarkably however, at stalled forks loss of PTIP *suppresses* nascent strand degradation in BRCA1 or BRCA2-deficient backgrounds, i.e. counteracts resection, and decreases fork-bound MRE11 103. Of note, loss of PTIP does not suppress fork reversal 43.

Understanding how PTIP performs its diverse functions at DSBs and stalled forks will require additional data. One possibility is that if PTIP facilitates recruitment of both MRE11 and Artemis at DSBs, it may thus restrain MRE11 at the stage of initiating resection. If so, this restraining function may be irrelevant at forks because, once again, the nick and gap-rich fork structure obviates the need for MRE11 as resection-initiator. On the other hand, Artemis may exacerbate nucleolysis at forks. *In vitro*, DNA-PKcs enables Artemis to cleave dsDNA 107, and it was shown to cleave stalled forks after prolonged arrest 108. An alternative explanation for PTIP’s role at forks is that it may be more important as a recruiter of the histone methyltransferase MLL3/4, which generates histone H3 trimethylated on Lysine 4.

RAD52 is known for its involvement in the HR-mediated DSB repair, and becomes critical for cell survival in the absence of BRCA1/2 (for review, see 109). *In vitro*, it can anneal ssDNA, facilitate strand exchange, and, under some conditions, stimulate RAD51 (*ibid*.) Remarkably, Mijic *et al*. showed that loss of RAD52 in BRCA2-deficient cells has a phenotype similar to PTIP loss: nascent strand resection at stalled forks is suppressed while fork reversal is unaffected. Moreover, without RAD52 less MRE11 is bound at stalled forks 43.

### Restrainers of RAD51

The list of proteins whose role involves upregulating or downregulating the level of RAD51 at stalled forks *in vivo* and/or fork substrates *in vitro* continues to grow (**Figure 3**). Some of these proteins directly act on RAD51, while the role of others is more indirect - for example, they may compete with RAD51 for the same substrates.

RECQ helicases RECQ5 and BLM were perhaps the first to be demonstrated to disassemble RAD51/ssDNA filament *in vitro* 110,111,112,113, albeit RECQ5 inhibits RAD51 strand invasion (D-loop), while BLM promotes it. Interestingly, RECQ5 may be recruited by MRE11 114. However, RECQ5 and BLM are not known to affect nascent strand degradation, suggesting that these proteins may be employed at clearing RAD51 during fork restoration and restart.

FBH1, an F-box-containing 3’-5’ DNA helicase, was also shown to displace RAD51 from ssDNA *in vitro* 115. Some *in vivo* activities of FBH1 are consistent with this role of a RAD51 restrainer: disruption of FBH1 has antirecombinogenic phenotype (*ibid.*), and also suppresses the phenotypes of two facilitators of fork protection, WRNIP and BOD1L (see below). However, FBH1 also reverses forks 38, thereby working in concert with RAD51, and promotes DSB formation at collapsed forks along with MUS81 17, thereby terminating the processes associated with fork stabilization and recovery. A recent work showed that FBH1 participates in ubiquitination of RAD51, which leads to its degradation 116. Assuming all these functions are distinct, further studies are needed to understand how they are selectively utilized in vivo.

PARI is another protein that can disrupt RAD51/DNA association *in vitro* and has anti-recombinogenic phenotype *in vivo* 117. A novel ssDNA binding protein, RADX, was identified recently as another restrainer of RAD51, which likely competes with RAD51 for ssDNA 55. Inactivation of RADX suppresses nascent strand degradation in BRCA2-deficient cells, suggesting that BRCA2/RADX double deficient cells may load enough RAD51 onto forks. RADX likely binds at reversed forks because suppression of reversal (by depletion of ZRANB3 or SMARCAL1) also suppresses phenotypes of RADX loss.

### Facilitators of RAD51

Constraints on RAD51 activity are counterbalanced by several proteins that either load RAD51 independently of BRCA2, or protect loaded RAD51. In Chinese hamster cells, deficiency in RAD51 paralogs RAD51C, XRCC2, or XRCC3 upregulates nascent strand degradation and exposes excess of parental DNA in single stranded form at stalled forks 118. Notably, deficiency in RAD51C and XRCC2 was additive with BRCA2 deficiency in nascent strand degradation, suggesting a BRCA2-independent contribution to RAD51 loading.

Fanconi Anemia proteins FANCD2 and FANCI likely enable RAD51 by protecting it on DNA. FANCD2 deficiency results in excessive nascent strand degradation at HU-arrested forks 61. *In vitro*, FANCD2/FANCI can stabilize RAD51/ssDNA filament and protect the ds/ss DNA junction behind the 3’ ssDNA overhang from nucleolysis 119. Work by Sobeck lab, using DNA polymerase inhibitor aphidicolin (APH) as means to arrest replication, suggests that the role of FANCD2 is also to control the activity of the structure-specific endonuclease/5’-3’ exonuclease FAN1 that is recruited to APH-arrested forks and can contribute to excessive resection of these forks in FANCD2-deficient cells 120. Interestingly, FAN1 recruitment to forks was facilitated by MRE11 and FANCD2. Thereby, FANCD2 contributes to both bringing a nuclease to forks and limiting nucleolytic attack on forks.

### Facilitators of fork protection

The following proteins contribute positively to fork protection, however, it is not yet clear, whether they facilitate RAD51 and/or restrain the nucleases.

In the absence of BOD1L, nascent strand degradation at forks stalled by HU is upregulated, as are the levels of parental ssDNA, albeit assayed using a different replication-stressing agent, mitomycin C 121,122. Upregulated nascent strand degradation without BOD1L is epistatic with BRCA1 or BRCA2 absence, suggesting that these genes are acting in the same pathway and/or on the same substrate. Furthermore, upregulated strand degradation (and ssDNA formation) upon BODL1-deficiency is partially suppressed by depletion of FBH1 and BLM, suggesting that BOD1L counteracts anti-RAD51 activity of these two proteins. Surprisingly however, depletion of DNA2 but not MRE11 or EXO1, suppresses the nascent strand degradation phenotype of BOD1L loss. BOD1L does not have known enzymatic activities.

Depletion of the ATPase WRNIP1 known for its association with WRN, generates a phenotype very similar to that of BOD1L with respect to its interactions with BRCA2 and FBH1 123. The level of parental ssDNA is also elevated in WRNIP1-depleted cells, though less RAD51 is bound to it. Remarkably however, unlike BOD1L, WRNIP1’s upregulated nascent strand degradation is suppressed by inactivation of MRE11. These findings may suggest that BOD1L and WRNIP1 belong respectively to DNA2/WRN-dependent and MRE11-dependent branches of fork resection (**Figures 3, 4**). Addressing this will require further research.

The list of proteins that influence RAD51 binding and/or modulate resection will likely continue to grow. Among new additions are REV1 124, which protects from nascent strand degradation and may be recruited by monoubiquitinated FANCD2; and the kinase NEK8 125. Another group of contributing proteins that should be mentioned are those that enable targeted degradation of the key participants, e.g. RAD51 126, WRN 127, EXO1 128, and others (reviewed in 129).

### How does it all come together? Toward an integrated view of fork remodeling

The overview of the proteins engaged in stalled fork remodeling suggests that many of them either contribute to both sides of the "standoff" between DNA protectors and DNA degraders, or are subject to counteracting activities of other proteins. In a system with so many moving parts, the exact balance between fork reversal, protection, and resection may depend on the sub-nuclear distribution, mutual ratios, and relative activity of the proteins involved, which may vary with the genetic and epigenetic background. The nature of the experimental perturbation may also play into this. For example, the level of RAD51 may influence how stringently fork reversal depends on it, while the degree of RAD51 depletion may affect its ultimate phenotype at forks. That is, only a complete loss of RAD51 may abolish fork reversal while partial depletion will only disrupt its function in fork protection.

It should be appreciated that most of the cell lines used in fork remodeling studies are transformed or cancer-derived, and fork remodeling in cancer cells may be already rewired or relaxed under the pressure of chronic replication stress. For example, lines HCT116 and U2OS exhibit baseline nascent strand degradation 73, unlike primary fibroblasts 72. Genomic analysis of the U2OS line found potentially non-neutral SNVs in several genes 130 and in particular, in AND-1/WDHD1, which has roles in replication and homologous recombination, and was shown to interact with many proteins including BRCA1, CtIP, and MRE11, and regulate resection 131,132. Genetic and epigenetic heterogeneity of our model systems should become not just an extra consideration as we face the necessity to organize the growing body of data, but also a resource for discovery as it becomes clear that the state of fork protection has translational significance as a determinant of cancer chemo-resistance, as we will discuss below.

## SURVIVAL AND CHEMO-RESISTANCE: FORM REMODELING ENTERS THE TRANSLATIONAL SPOTLIGHT 

Lethality of BRCA1 or BRCA2 deficiency when combined with PARP1 inhibition or cisplatin is perhaps the most famous of synthetic lethal interactions discovered 133 and translated into the clinic as treatment against breast and ovarian cancer 134,135. Yet, resistant tumors arise frequently, which prompted efforts by many labs to identify the determinants of resistance 136. Research focused on PARP1 inhibitor olaparib in the context of breast and ovarian cancer uncovered multiple pathways of partial or complete resistance that were utilized by cancer cells. In many cases resistance-causing mutations reversed HR defect of parental BRCA1 or BRCA2-deficient cells, but remarkably, in many other cases they restored stalled fork protection while leaving HR defective. In fact, *in vivo* and *in vitro* selection for cisplatin or olaparib resistance in BRCA2 or BRCA1-deficient backgrounds readily yields tumors or cell line clones in which the parental nascent strand degradation at forks is now suppressed (see refs. below). These important findings brought replication fork remodeling to the frontline of cancer chemotherapy. Collectively, the data point at the equilibria between fork reversal and restoration and between resection and protection as "breeding grounds" of chemo-resistance. However, intensifying research by many labs also poses additional questions about the nature of the lethality of PARP1 inhibition in BRCA-deficient cells, and about the role of PARP1 in nascent strand degradation.

### The PARP1 paradox

Excessive nascent strand degradation correlates with increased DNA breakage 60,61. Thus, it can be hypothesized that the reduced viability of BRCA-deficient cells subjected to PARP1 inhibitor (PARPi) can be at least in part mediated by the highly elevated nascent strand degradation. Indeed, some studies found that PARP1 inhibition by olaparib exacerbated MRE11-dependent nascent strand degradation at stalled forks in BRCA2-deficient cells 137 (**Table 2**). Also in agreement with this hypothesis is the observation that PARPi resistance in BRCA2-deficient cells can emerge due to secondary mutations that also suppress nascent strand degradation 103 (also, see next section). However, using different cell sources as well as combinations of gene knockouts, RNAi, or olaparib, several studies now have come to a different conclusion (**Table 2**): PARP1 deficiency or inhibition *suppress* nascent strand degradation and reduce MRE11 binding to forks in BRCA2-deficient cells 43,138,139. Similarly, Chaudhuri *et *a.l observed that *PARP1-/-,* *BRCA1-/-* double knockout mouse B cells exhibited a suppressed nascent strand degradation compared to *BRCA1-/-* cells 103.

**Table 2:** TABLE 2. Nascent strand degradation phenotypes of BRCA-deficient, PARP1-deficient cells. Abbreviations: OLA, olaparib, K.O., knockout, RNAi, siRNA or shRNA-mediated depletion, HU, hydroxyurea.

**Perturbation of BRCA2/1 (first unless stated otherwise)**	**Perturbation of PARP1(second or concurrent unless stated otherwise)**	**Cells**	**Nascent strand degradation phenotype**	**Clonogenic survival**	**MRE11 recruitment**	**Ref**
BRCA2-/-	OLA (concurrent with HU)	V-C8 CHO	**exacerbated **w/o BRCA2, also in BRCA2-complemented cells	Suppressed (w HU)	reduced (nuclear foci)	140
BRCA2-/- **(second)** Conditional K.O.	OLA **(first)**, RNAi **(first)**, PARP1-/- K.O. **(first)**	mouse ESC, mouse B cells	suppressed	Yes (w/o HU)	reduced at forks (iPOND)	138
RNAi **(second)**	RNAi **(first)**	MCF10a	suppressed	NA	NA	138
BRCA2-/- Conditional K.O.**(second)**	PARP1-/- K.O. **(first)**	mouse B cells	suppressed	Yes (w/o HU)	NA	103
RNAi	OLA (prior to HU)	U2OS	suppressed	NA	NA	43
BRCA2 delEx3-4/-; BRCA2-/-	OLA , RNA, K.O.	MCF10a	suppressed	Low/absent (w/o HU)	reduced on chromatin	139

These findings are more in line with the consensus that PARP1 facilitates recruitment of MRE11 to stalled forks 76,141, and call for a more nuanced view of the relationship between PARP1 and BRCA proteins at replication forks. For example, PARP1 deficiency may block nascent strand degradation by blocking fork reversal, similar to the effect of the loss of SMARCAL1, ZRANB3, or HLTF 28,43,58. If so, failure to reverse forks may in and of itself trigger DNA damage in the context of replication stress in BRCA-deficient cells, as described in previous sections. Indeed, Mijic *et al*. observed a mild increase in chromatid breaks and gaps after forks stalling in BRCA2-depleted cells treated with olaparib 43. Combining ZRANB3 knockout with BRCA2 depletion also increased chromatid breaks compared to cells with only one of these two genes inactivated, and PARPi was epistatic with the ZRANB3 deficiency. However, Taglialatela *et al*. found that chromosomal aberrations and DNA damage were reduced upon depletion of SMARCAL1 or ZRANB3 in BRCA1 or BRCA2-depleted cells 44. Overall, further research is required to elucidate the complex relationships between PARP1, BRCA proteins, fork remodeling processes, and DNA damage response and cell survival. Some potential areas of consideration are suggested below.

First, the unique nature of PARP1 loss may be in that it causes inappropriate *restoration* of reversed forks rather than blocking reversal in the first place, as the SNF2 family members or RAD51 do. Second, at least for some cases, trapping of PARP1 on DNA by olaparib may explain how a differential phenotype may emerge from PARP1 inhibition versus depletion in BRCA2-deficient cells 142. Third, suppression of fork reversal upon PARP1 loss may well have a dual effect: at a high level of fork stalling (due to exogenous stressors like HU) failure to reverse forks is deleterious, while at a low level of fork stalling it may speed up S phase at the cost of decreased genomic stability. This logic may explain why PARP1 loss actually supports proliferation of BRCA2-deficeint non-cancer cells 103,138, given that these cells display constitutive fork progression problems 42,142 that trigger MUS81-mediated fork collapse and mitotic DNA synthesis 99,139. Forth, the effect of PARP1 (like that of RAD51) may depend on whether it is a significant contributor to fork reversal in a given cell background, given that fork remodeling and DNA damage response may have undergone rewiring under selective pressure.

It should also be noted that the variability of PARP1 phenotypes may reflect its multiple, often counteracting inputs at the fork. For example, PARylation facilitates recruitment of BRCA1 to DSBs 143 and may do so at stalled forks. PARP1 also facilitates recruitment of NHEJ factors XRCC1 and DNA-PKcs to stalled and unresected forks 140. Ku70/80 heterodimer binds to stalled forks, likely, at the end of a reversed arm that mimics a one-ended DSB, and protects it from the nucleases. Ku70/80 together with DNA-PKcs mediate timely fork restart 140,144,145,146. These data are consistent with the expectation that PARP1 inhibition should upregulate resection. However, activated PARP1 also reduces Ku70/80 affinity for DNA ends (reviewed in 147, which would be consistent with PARP inhibition suppressing resection.

### Alterations that confer chemo-resistance to BRCA-deficient cells correlate with suppressed nascent strand degradation

Perhaps the best testimony in favor of the association between fork protection and chemo-resistance is the accumulation of data that demonstrate this association in diverse, independently derived genetic backgrounds. For example, overexpression of FANCD2 was shown to suppress nascent strand degradation and confer PARPi resistance in a subset of BRCA1 or BRCA2-deficient cases 148. Also, in BRCA1-deficient ovarian cancer cells, RAD51 paralogs XRCC2 and XRCC3 are capable of loading enough RAD51 onto stalled forks (in an ATR-dependent manner), thus providing proto-resistance to PARPi that can evolve to full resistance 149. Increased loading of RAD51 at forks is also consistent with the olaparib-resistant phenotype of RADX loss 55. PTIP, which affects MRE11 level at stalled forks, is another gene whose loss restores olaparib resistance and suppresses nascent strand degradation of BRCA1 and BRCA2-deficient cells 103. This activity of PTIP depends on its ability to associate with MLL3/4, a histone methyltransferase that methylates histone H3 on Lysine 4 150, and indeed Lysine 4 mono- and trimethylated H3 (H3K4me3) accumulates at resected forks in PTIP-proficient and not PTIP-deficient cells. Moreover, loss of MLL3/4 partially suppresses nascent strand degradation in BRCA2-deficient cells.

Discovery of the involvement of MLL3/4 implicates epigenetic regulation by histone modification in fork protection, adding another layer of complexity to the already elaborate system. Another prominent epigenetic regulator found to affect nascent strand degradation is CHD4, a member of the NuRD complex involved in gene regulation via nucleosome remodeling and histone deacetylation 151. CHD4 depletion enhanced resistance to olaparib and cisplatin in BRCA2-deficient but not in BRCA1-deficient cells 152, and suppressed nascent strand degradation in BRCA2-deficient cells 103. Lastly, a very recent study uncovered a similar role for the member of the Polycomb Repressive complex 2, EZH2, a histone H3 Lysine 27 methyltransferase, in BRCA2-deficient breast and ovarian cancer cells. Inhibition of EZH2 conferred PARPi and cisplatin resistance to these cells and suppressed nascent strand degradation at stalled forks 153 (reviewed in 154). Intriguingly, the authors traced the effect of EZH2 to its ability to mediate recruitment of the endonuclease MUS81 rather than RAD51 or MRE11 to stalled forks, and showed that downregulation of EZH2 protects forks by *suppressing* recruitment of MUS81. Overall, our understanding of the epigenetic control of fork protection is far from complete, but it is safe to assume that it will reveal further heterogeneity and plasticity of fork protection mechanisms of cancer cells.

## FORK REMODELING AND INNATE IMMUNITY 

In this last section we would like to bring attention to a developing, exciting area of research that may well improve our understanding of the effects the deregulation of fork remodeling has on cancer and normal cells. This area is innate immune response.

Briefly, innate immune response is a universal cell-intrinsic response to bacteria and viruses. One of its jobs is to recognize and react to foreign DNA found in the cytoplasm, which involves multiple sensor molecules. Over the past decade, studies have begun to make connections between DNA damage response and the immune response to self-DNA 155,156 mediated by a cytosolic DNA sensor cGAS and a downstream adaptor STING (for review 157). The crucial discovery that emerged is that endogenous short genomic DNA species produced in the course of checkpoint activation and DNA repair can trigger innate immunity if not properly degraded, and that this may contribute to the development of such autoimmune diseases as systemic lupus erythrematosus (SLE) and Aicardi Goutieres Syndrome (AGS) 158,159,160,161. TREX1, a major single-stranded DNA exonuclease, has emerged as a key controller of the abundance of short DNA species, both single- and double-stranded, that are "shed" by the damaged and repairing genome. Without TREX1, both DNA damage checkpoint signaling and type I interferon (IFN) production (a hallmark of innate immune response) are upregulated 162,163,164. Moreover, TREX1 controls abundance of short ssDNA species upon replication fork stalling by hydroxyurea 165.

Several studies have now made explicit associations between DNA resection during DNA repair or fork stalling and innate immune response to self-DNA. Erdal *et al*. detected a spike in short cytoplasmic ssDNA in cells treated with ionizing radiation (IR), mitomycin C or cisplatin, which activated innate immune response. Depletion of resection nucleases DNA2, EXO1, and BLM, but, interestingly, not MRE11 and CtIP, suppressed these responses, and TREX1 deficiency exacerbated them 166. The authors also showed that inhibition of PARP1 boosted both basal and IR-induced levels of cytosolic ssDNA. Notably, TREX1 was found to associate with PARP1 in the nucleus upon DNA damage 167. Non-involvement of MRE11 and CtIP may suggest that only long-range resection produces reactive ssDNA species. On the other hand, MRE11 168 as well as Ku70/80 169 were identified as dsDNA sensors in the cytoplasm that feed into the STING pathway. This role of MRE11 may, in theory, complicate interpretation of innate immune reaction to the depletion of the protein.

Pasero and colleagues identified SAMHD1 as a cofactor of MRE11 at stalled forks. SAMHD1 is a dNTPase with roles in cell-intrinsic antiviral response, and one of the genes mutated in AGS 170. SAMHD1 was also shown to promote resection with MRE11 and CtIP at DSBs 171. Pasero lab showed that deficiency in SAMHD1 negatively affects both fork restart and activation of the S phase checkpoint. Remarkably, it also upregulates release of resected DNA fragments into the cytoplasm where they trigger STING-dependent type I interferon response (P. Pasero, pers. comm.) Thus, SAMHD1 is one of the links between free DNA, checkpoint signaling, and inflammation.

In another study, RAD51 depletion was correlated with elevation of cytoplasmic ss and dsDNA, upregulation of innate immune response including STING, and enhanced nascent strand degradation at forks after IR 74. All of these phenotypes were suppressed by inhibition of MRE11 exonuclease activity. An interesting addition to this line of evidence was generated by Wolf *et al*. 172, where the authors demonstrated that failure to clear free short ssDNA can take a toll on fork remodeling. The authors exploited a TREX1-deficient background to demonstrate that excess of short free ssDNA that accumulates in these cells is bound by RPA and RAD51 both in the cytosol and the nucleus. More remarkably, they also provided evidence suggesting that this condition can deplete the pools of RPA and RAD51 available for binding to genomic DNA at sites of damage and fork stalling, and thus can compromise DNA repair and fork protection. Overall, the data described above firmly establish innate immune response as part of a cellular stress response associated with deregulation of resection of stalled replication forks. Moreover, by affecting the availability of proteins like RAD51, RPA, and MRE11, innate immune response to replication stress-associated self-DNA may become another determinant of cancer susceptibility to chemotherapy.

## CONCLUDING REMARKS 

We hope to have shown that fork remodeling mechanisms emerge as balancing acts of many counteracting processes - a game of substrates that revolves around degradation versus protection of nascent DNA strands, and whose original main characters, MRE11 and RAD51, are surrounded by a whole cast of others. Moreover, fork remodeling moves into the spotlight as a major contributor to carcinogenesis, chemotherapy resistance, and inflammation.

We have seen that many HR and NHEJ proteins are involved in fork protection and curiously, often in a somewhat "off-label" way that is not exactly identical to their roles in DNA repair. We have also seen that close inspection of fork remodeling reveals cell line-dependent heterogeneity and malleability under selective pressures of carcinogenesis. While complete understanding of these complexities is still farther down the road, a few general considerations may guide us on the way.

First, it is worth remembering that fork remodeling phenotypes, as typically measured, are likely an average of heterogeneous responses to fork stalling in different genomic and epigenomic regions, next to actively transcribed genes or not, with different configurations of parental and daughter strands (ssDNA gaps, nicks, 5’ vs. 3’ overhangs), for different periods of time since the onset of fork-arresting treatment; and in early versus late S phase of the cell cycle. Aggregated together, these responses may create an impression of one too many proteins contributing semi-redundantly, while in reality cells may use "local" solutions to fork remodeling with only a subset of proteins involved in each case. To bring just one example, only a particular subset of stalled forks - those with leading strand gaps - may actually be reversed by SMARCAL1 37. Our ability to examine homogeneous subpopulations of cells and forks will be increasingly important in the future.

Second, it should be considered that the phenotypes often measured in perturbation studies of fork protection may not be a simple consequence of a protein’s absence, but a re-balancing act that the whole network undergoes because of it, i.e. an absence of function of one may actually cause a gain of function of several others.

And lastly - even if variability is widespread, the functional variables that define how fork remodeling is configured in cells are still few, and common. These are: 1) robustness and efficiency of loading of RAD51 at forks and the extent of its dependency on BRCA1/2; 2) robustness and dominance of fork reversal over fork restoration; 3) robustness and efficiency of nucleolytic activity at forks. The additional variables that are less studied at present but may come to the fore in the future are: efficiency of loading of NHEJ proteins as protective factors, the degree of nucleosome mobility and histone turnover at forks, and the efficiency of innate immune response. As the data show, while chemotherapeutic resistance in the fork protection-defective cancer cells arises frequently, it seems to work by adjusting the above-mentioned common variables. As such it is predictable - and thus can be preempted.

## Abbreviations

ds: ouble-stranded
DSB: double strand break
HR: homologous recombination
HU: hydroxyurea
IR: ionizing radiation
NHEJ: non-homologous end joining
ss: ingle-stranded
